# TRAIL-R2 Superoligomerization Induced by Human Monoclonal Agonistic Antibody KMTR2

**DOI:** 10.1038/srep17936

**Published:** 2015-12-17

**Authors:** Taro Tamada, Daisuke Shinmi, Masahiro Ikeda, Yasushi Yonezawa, Shiro Kataoka, Ryota Kuroki, Eiji Mori, Kazuhiro Motoki

**Affiliations:** 1 Quantum Beam Science Center, Japan Atomic Energy Agency, 2-4, Shirakata, Tokai, Ibaraki 319-1195, Japan; 2 Research Core Function Laboratories, R&D Division, Kyowa Hakko Kirin Co., Ltd., 3-6-6, Asahi-machi, Machida, Tokyo, 194-8533, Japan; 3 Immunology & Allergy Research Laboratories, R&D Division, Kyowa Hakko Kirin Co., Ltd., 3-6-6, Asahi-machi, Machida, Tokyo, 194-8533, Japan; 4 Business Development Department, Kyowa Hakko Kirin Co., Ltd., 1-6-1, Ohtemachi, Chiyoda-ku, Tokyo, 100-8185, Japan; 5 R&D Planning Department, R&D Division, Kyowa Hakko Kirin Co., Ltd., 1-6-1, Ohtemachi, Chiyoda-ku, Tokyo, 100-8185, Japan; 6 Oncology Research Laboratories, R&D Division, Kyowa Hakko Kirin Co., Ltd., 3-6-6, Asahi-machi, Machida, Tokyo, 194-8533, Japan

## Abstract

The fully human monoclonal antibody KMTR2 acts as a strong direct agonist for tumor necrosis factor-related apoptosis-inducing ligand (TRAIL) receptor 2 (TRAIL-R2), which is capable of inducing apoptotic cell death without cross-linking. To investigate the mechanism of direct agonistic activity induced by KMTR2, the crystal structure of the extracellular region of TRAIL-R2 and a Fab fragment derived from KMTR2 (KMTR2-Fab) was determined to 2.1 Å resolution. Two KMTR2-Fabs assembled with the complementarity-determining region 2 of the light chain via two-fold crystallographic symmetry, suggesting that the KMTR2-Fab assembly tended to enhance TRAIL-R2 oligomerization. A single mutation at Asn53 to Arg located at the two-fold interface in the KMTR2 resulted in a loss of its apoptotic activity, although it retained its antigen-binding activity. These results indicate that the strong agonistic activity, such as apoptotic signaling and tumor regression, induced by KMTR2 is attributed to TRAIL-R2 superoligomerization induced by the interdimerization of KMTR2.

Tumor necrosis factor (TNF)-related apoptosis-inducing ligand (TRAIL/Apo2L)^1^,^2^ induces apoptosis in a wide variety of human cancer cell lines, but not in most normal human cells^3^,^4^. TRAIL activates two distinct receptors, TRAIL-R1 (DR4)^5^ and TRAIL-R2 (DR5)^6^,^7^,^8^, both of which possess a death domain (DD) in their cytoplasmic tail that can interact with the apoptotic machinery. The assembly and trimerization of TRAIL-R1 and TRAIL-R2 are prerequisites for transducing an apoptosis signal^9^,^10^. The structure of the extracellular region of TRAIL-R2 (ecTRAIL-R2) in complex with TRAIL has shown that similar to other members of the TNF and TNF receptor superfamily, a TRAIL trimer binds to three receptors^11^,^12^,^13^, suggesting that a trimeric ligand–receptor complex is the functional unit for signaling^14^. This trimeric complex has also been determined for ternary (3:3:3) complex with Fab fragment derived from AMG 655 which increases antitumor activity in cooperation with TRAIL^15^. In addition, the structures of ecTRAIL-R2 have been determined for a 2:2 complex with glycoprotein UL141 of human cytomegalovirus^16^ and for 1:1 complexes with phage-derived Fabs, YSd1^17^, BDF1^18^, and Apomab^19^.

Bivalent IgG antibodies normally require cross-linking for further oligomerization to mimic natural ligands. Cross-linking of antibodies with certain reagents, such as secondary antibodies, has been reported to enhance their antitumor effects. For example, Griffith *et al.*^20^ and Chuntharapai *et al.*^21^ reported that the antitumor effects of murine antibodies to TRAIL-R1 and TRAIL-R2 were strongly enhanced in the presence of goat anti-mouse IgG, which aided in antibody cross-linking. We previously reported the generation of several human monoclonal antibodies specific for TRAIL-R1 or TRAIL-R2 that were derived from transchromosomal mice (KM mouse)^22^ expressing human immunoglobulin repertoires^23^. We generated a novel anti-TRAIL-R2 monoclonal antibody, KMTR2, which acts as a direct agonist possessing the ability to induce apoptosis without cross-linking^24^.

KMTR2 can oligomerize soluble TRAIL-R2 fused with the Fc region of IgG (ecTRAIL-R2:Fc) and clusters membrane TRAIL-R2 on cell surfaces without cross-linking, inducing the death of human tumor cells *in vitro* and established tumors *in vivo*^24^. Furthermore, treating tumor-bearing nude mice with KMTR2 significantly suppressed the growth of subcutaneous glioma xenografts and resulted in complete regression^25^. Therefore, specifically targeting the death receptor pathway through TRAIL-R2 using direct agonistic antibodies may provide a novel therapeutic strategy for malignant tumors.

Here we report on the tertiary structure of KMTR2-Fab in complex with ecTRAIL-R2 and describe the mechanism of how this direct agonistic antibody accelerates TRAIL-R2 oligomerization. Based on its crystallographic symmetry, the dimeric complex structure of KMTR2 appeared to be a functional unit for generating a superoligomeric complex. Interference of this interface with an Asn53 to Arg mutation in the light kappa chain of KMTR2 resulted in the loss of the direct agonistic activity but not the binding activity of KMTR2, indicating that interdimerization of KMTR2 is responsible for TRAIL-R2 superoligomerization, leading to the strong agonistic activity of KMTR2.

## Results

### Overall structure of ecTRAIL-R2 in complex with KMTR2-Fab

**Figure 1:**
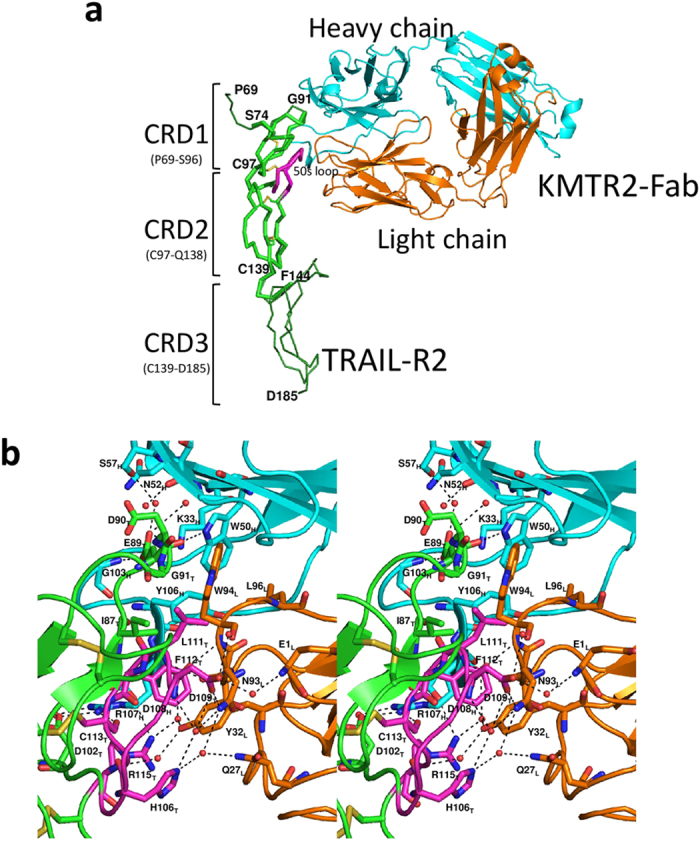
Crystal structure of the 1:1 complex between ecTRAIL-R2 and KMTR2-Fab. (**a**) Overall structure. The TRAIL-R2 molecule and the heavy and light chains of KMTR2 are colored green, cyan, and orange, respectively. A 50s loop and disulfide bonds in TRAIL-R2 are indicated by magenta and a stick model, respectively. The TRAIL-R2 structure in complex with TRAIL (PDB ID: 1D4V, dark green) is superimposed on a KMTR2 complex. (**b**) Close-up view of the interface between ecTRAIL-R2 and KMTR2-Fab (stereo representation). The orientation of this figure is the same as in (**a**). Residues related to antigen–antibody binding are represented by a stick model. Hydrogen bonds are indicted by dashed lines. Subscripts H, L, and T indicate KMTR2 heavy and light chains and TRAIL-R2, respectively.

The tertiary structure of ecTRAIL-R2 in complex with KMTR2-Fab was determined to 2.1 Å resolution by X-ray crystallography (Fig. 1a). Although the electron densities of the N-terminal (Ala54–Arg73) and C-terminal (Arg145–Lys181) regions in ecTRAIL-R2 were unclear, the final model included two cysteine-rich domains (CRD1: Ser74–Ser96 and CRD2: Cys97–Gln138), and some part of CRD3 (Cys139–Phe144) of TRAIL-R2 (Fig. 1a) and the heavy (Gln1–Ser226, excluding Arg142–Ser145) and light (Glu1–Cys214) chains of KMTR2-Fab. This model also included one N-linked sugar chain bound at Asn73 of the KMTR2 heavy chain and five glycerol molecules, one chloride ion, and 276 water molecules. The overall structure of ecTRAIL-R2 in complex with KMTR2-Fab was similar to that in complex with TRAIL (PDB ID: 1D4V)^11^, with an RMSD value of 1.02 Å for all main chain atoms (Fig. 1a). Of note, the position of Gly91 in the KMTR2-Fab complex was flipped against that in a TRAIL complex.

The tertiary structure of KMTR2-Fab without ecTRAIL-R2 was also determined to 2.5 Å resolution. Although the electron densities of the central part (Ser102–Tyr106) of complementarity-determining region (CDR) 3 of the heavy chain was unclear, the final model included the heavy (Gln1–Ser226, excluding Ser102–Tyr106 and Ser145–Glu146) and light (Glu1–Cys214) chains, one N-linked sugar chain at Asn73 of the KMTR2 heavy chain, two sulfate and three chloride ions, and 42 water molecules. The overall structure of KMTR2-Fab was conserved regardless of antigen binding, with an RMSD value of 1.17 Å for all main chain atoms (Fig. S1).

### TRAIL-R2/KMTR2-Fab interface

KMTR2-Fab recognized CRD1 and CRD2 of TRAIL-R2 in the direction perpendicular to TRAIL-R2 (Fig. 1a). The area of the contact region between TRAIL-R2 and KMTR2-Fab was calculated to be 623 Å^2^, with contributions of 322 Å^2^ from the heavy chain and 301 Å^2^ from the light chain of KMTR2. To determine the epitope recognition scheme of KMTR2, residues located within a distance of 4.0 Å between TRAIL-R2 and KMTR2-Fab were determined (summarized in Table 1). KMTR2-Fab recognized two epitopes (epitope-1 and -2) in ecTRAIL-R2 (Fig. 1a). Epitope-1 included Ile87, Glu89, Asp90, and Gly91 in CRD1, which was mostly recognized by CDR1–3 of the heavy chain (Fig. 1b, Table 1). The side chain Glu89 atoms of TRAIL-R2 interacted with Lys33 in CDR1, Gly103 in CDR3, and Trp50, Asn52, Ser57 in CDR2 in the heavy chain of KMTR2 via direct and water-mediated hydrogen bonds. The aromatic Trp94 ring in CDR3 of the light chain exhibited a stacking interaction parallel to the peptide surface of Asp90–Gly91 of TRAIL-R2, which was the greatest positional difference compared with that in a TRAIL complex (Fig. 1a). The side chain (Cβ atom) of Trp94 in CDR3 of the light chain was also positioned within a distance of 4.0 Å against the side chain atoms of Ile87 in epitope-1.

Epitope-2 comprised Asp102 and some residues in a “50s loop” (Thr105–Arg115) in CRD2, one of the two natural ligand recognition sites, and interacted with CDR1 and CDR3 of the light chain and CDR3 of the heavy chain (Fig. 1b, Table 1). A hydrogen bond network was generated between epitope-2 (His106 and Asp109) and the light chain of KMTR2 (Gln27 in CDR1, Asn93 and Trp94 in CDR3, and Glu1). The Arg107 side chain in CDR3 of the heavy chain intruded into the 50s loop of TRAIL-R2 and participated in recognizing epitope-2 (Asp102, Phe112, Cys113, and Arg115) by hydrogen bonding and van der Waals interactions. The hydroxyl oxygen atom of the Tyr32 side chain in CDR1 of the light chain interacted with the main chain amide and side chain guanidino group of Arg115 in the 50s loop via water-mediated hydrogen bonds. The side chains of Leu111 and Phe112 were wedged between two tyrosine residues (Tyr32 in CDR1 of the light chain and Tyr106 in CDR3 of the heavy chain), which provided van der Waals interactions. The side chain atoms of Leu111 were also located within a distance of 4.0 Å against three residues (Asn93, Trp94, and Lue96) in CDR3 of the light chain.

We compared the structures described above with other TRAIL-R2 structures in complex with other Fab fragments. In addition to the KMTR2-Fab structure, the crystal structures of phage-derived Fab fragments, YSd1 (PDB ID: 1ZA3)^17^, BDF1 (PDB ID: 2H9G)^18^, and Apomab (PDB ID: 4OD2)^19^, were previously determined. Apomab exhibits some agonistic activity (EC_50_: approximately 100 ng/ml) without using a cross-linking reagent^19^. Recently, the crystal structure of Fab fragment derived from AMG 655 was reported (PDB ID: 4N90)^15^. AMG 655 has no activity in the absence of cross-linking, but increases it in cooperation with TRAIL.

KMTR2 recognized the N-terminal region of TRAIL-R2 with a contact area of 623 Å^2^ (Fig. S2a) that involved both epitope-1 and -2, which was less than half of the contact area (1427 Å^2^) of the interface of a 3:3 complex of TRAIL and TRAIL-R2 (Fig. S2b). Epitope-2 for KMTR2 involved the 50s loop region, which was a TRAIL recognition site in CRD2. Apomab interacted with the C-terminal region of ecTRAIL-R2, which was nearly perpendicular to that recognized by KMTR2 (Fig. S2c). Epitope recognition by Apomab did not have any overlaps in CRD1 and CRD2 with that by KMTR2, but had a little overlap with the TRAIL recognition site, a 90s loop, in CRD3. AMG 655 interacted with ecTRAIL-R2 in the same direction of recognition by Apomab (Fig. S2b). Epitope recognition by AMG 655 mostly overlapped in CRD3 with that by Apomab. In contrast, epitope recognition by BDF1 and YSd1, which have little or no agonistic activity^19^, predominantly overlapped in CRD1 and CRD2 with that by KMTR2 (Fig. S2d, e). The contact area (623 Å^2^) between TRAIL-R2 and KMTR2 was smaller than those with YSd1 (715 Å^2^), BDF1 (851 Å^2^), and Apomab (805 Å^2^). Thus, this suggested that antigen recognition by CRD1 and CRD2 was insufficient to determine the direct agnostic activity of KMTR2.

### Interaction between two KMTR2-Fab fragments in crystal packing

Next, we focused on the crystal packing of ecTRAIL-R2/KMTR2-Fab complex because an oligomer configuration rendered by crystallographic symmetry often corresponds to the following biological assemblies. For a 1:1 complex structure between ecTRAIL-R2 (PDB ID: 1D4V), a 3:3 configuration via three-fold crystallographic symmetry reflects an activated TRAIL-R2 state. Based on two-fold crystallographic symmetry, we have also elucidated a 2:2 structure for the signaling complex between granulocyte colony-stimulating factor (GCSF) and its receptor (GCSF-R), with reference to several mutational analyses^26^.

**Figure 2:**
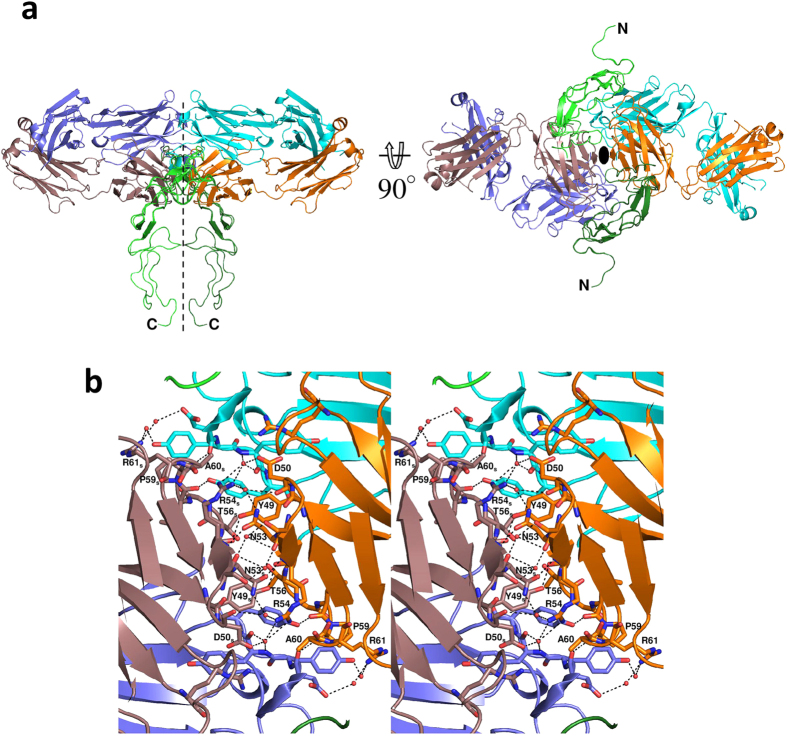
Dimeric structure of an ecTRAIL-R2/KMTR2-Fab complex rendered by crystallographic two-fold symmetry. The heavy and light chains of neighboring molecules rendered by crystallographic symmetry are colored purple and brown, respectively. (**a**) Overall structure. Dashed lines and ellipse indicate the two-fold axis. (**b**) Close-up view of the interface between two KMTR2-Fab molecules (stereo representation). Residues related to the interaction between two KMTR2-Fab molecules are represented by a stick model. The subscript “S” in label means “symmetry” molecule.

The 2:2 complex structure between ecTRAIL-R2 and KMTR2-Fab rendered by crystallographic symmetry is shown in Fig. 2a (unobserved CRD3 structure in TRAIL-R2 was constructed by superimposition with another TRAIL-R2 structure). Two KMTR2-Fab molecules interacted with CDR2 of the light chain via two-fold crystallographic symmetry, and consequently, two TRAIL-R2 molecules were situated in parallel within a C-terminal distance of 20 Å. It is believed that this adjacent configuration of extracellular regions results in access to each DD in the intracellular regions of TRAIL-R2 molecules, leading to activation of a caspase-dependent apoptotic pathway^6^.

Table 2 lists the contact pairs within 4 Å (3.6 Å for detecting hydrogen bonds) between two KMTR2-Fab fragments. The contact region mainly consisted of the region from Tyr49 to Arg61 located in CDR2 of the light chain, including six direct hydrogen bonding interactions and four van der Waals interactions, which may have partly accounted for the strong interaction between two KMTR2-Fab fragments (Fig. 2b). In particular, Arg54 in CDR2 of the light chain primarily contributed to the interaction between two KMTR2-Fab fragments, with three direct and five water-mediated hydrogen bonds.

### Binding activities of KMTR2 and its LkN53R mutant

We assumed that Fab dimerization based on this two-fold symmetry was essential for the direct agonistic activity of KMTR2. To confirm whether the interaction between two KMTR2-Fab fragments via crystallographic symmetry was essential for KMTR2 apoptotic activities, we attempted to incorporate a bulky residue into this interface to disrupt this interaction. We designed a mutant, Asn53 to Arg, in the light kappa chain of KMTR2 (LkN53R) to prevent dimerization at this site. Because Asn53 in CDR2 of the light chain was located near the two-fold axis between the two KMTR2-Fab fragments, the Asn53 to Arg mutation should have resulted in introducing both steric hindrance and charge repulsion from the associations of four positive charges, two from Arg53 after mutation and two from Arg54 (Fig. 2b).

**Figure 3:**
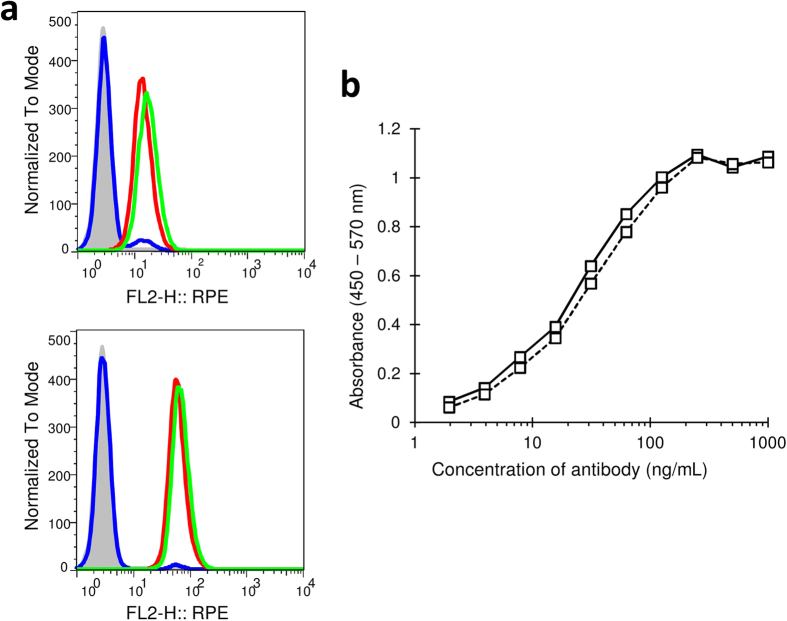
Binding activities of KMTR2 and its mutant (LkN53R) to TRAIL-R2. (**a**) Flow cytometry results. Antibody concentrations are 100 ng/mL (upper) and 1000 ng/mL (lower). X-axis indicates R-phycoerythrin (RPE) fluorescence signal intensity. Data for KMTR2, LkN53R mutant, and aDNPG1 (negative control) are shown as green, red, and blue lines, respectively. Gray-colored histogram is the control (2^nd^-PE). (**b**) ELISA results. Data for KMTR2 and the LkN53R mutant are shown as solid and dashed lines, respectively. Both in the flow cytometry and ELISA experiments, independent experiments were performed three times, and a representative result is shown, respectively.

We first examined the effect of this mutation on the binding activity with TRAIL-R2. As determined by flow cytometry analysis, both KMTR2 and its LkN53R mutant specifically bound to endogenous TRAIL-R2 in Colo205 cells (Fig. 3a). The fluorescence signal intensity of KMTR2 was similar to that of the LkN53R mutant at concentrations of both 100 ng/mL and 1000 ng/mL. The signal intensities of both KMTR2 and the LkN53R mutant increased in a concentration-dependent manner. In addition, ELISA analysis showed that both KMTR2 and the LkN53R mutant resulted in similar dose-dependent increases in absorbance after binding to ecTRAIL-R2 fused with Fc and reached saturation at an antibody concentration of 200 ng/mL (Fig. 3b). These results indicated that the Asn53 to Arg mutation had little effect on binding activity.

### Effect of the LkN53R mutation on TRAIL-R2 oligomerization

**Figure 4:**
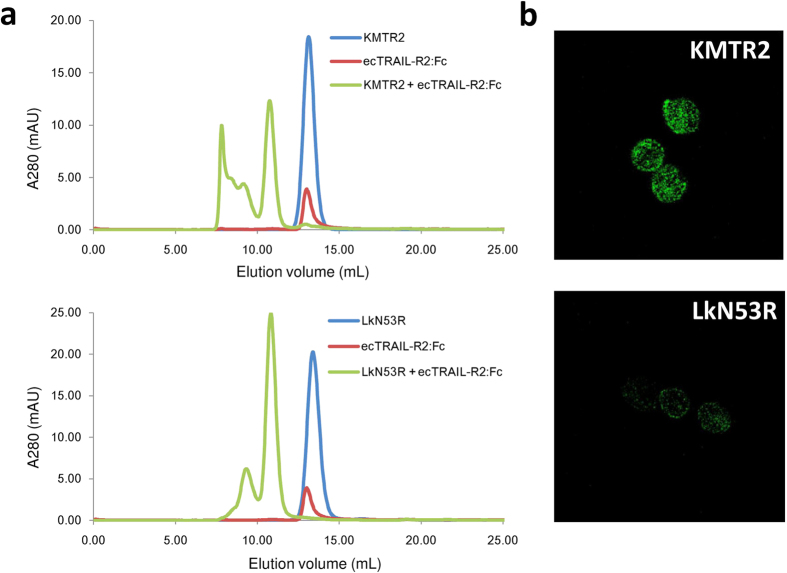
Immune complex analysis of TRAIL-R2 and monomer antibody. (**a**) A size exclusion chromatogram. KMTR2 (upper) or the LkN53R mutant (lower) was mixed with the ecTRAIL-R2:Fc fusion protein at a molar ratio of 1:1 and loaded onto Superdex 200 10/300 GL gel filtration column. Monomer antibodies and ecTRAIL-R2:Fc were also loaded independently. (**b**) Cell surface TRAIL-R2 immune complexes on Colo205 cells visualized by confocal microscopy (×40). The cells were incubated with monomer KMTR2 (upper) and the LkN53R mutant (lower) at 37°C for 1.5 h and then labeled with Alexa488.

Next, we examined the effect of this mutation on TRAIL-R2 oligomerization. Size exclusion chromatography (SEC) showed that KMTR2 can oligomerize ecTRAIL-R2:Fc (Fig. 4a, upper). The immune complex (ecTRAIL-R2:Fc/KMTR2) was eluted in the void volume fraction. In contrast, the ecTRAIL-R2:Fc/LkN53R complex was eluted within 250 kDa (Fig. 4a, lower), which was estimated by reference to the previous SEC-light scattering analysis^24^. The formation of immune complexes on the surface of Colo205 cells was visualized by confocal microscopy. Consistent with the idea that KMTR2 induces receptor clustering, Colo205 cells incubated with KMTR2 formed specific patches (Fig. 4b, upper). In contrast, the LkN53R mutant showed dispersed staining on Colo205 cells (Fig. 4b, lower). These results indicated that the LkN53R mutation abolished the higher oligomerization of TRAIL-R2.

### Apoptosis-inducing activities of KMTR2 and LkN53R

**Figure 5:**
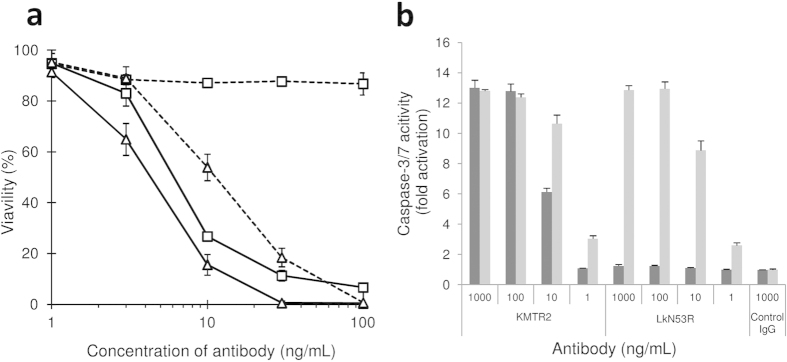
Apoptosis-inducing activity of KMTR2 and the LkN53R mutant. (**a**) Dose-dependent responses of apoptosis-inducing activity by a purified monomer fraction of KMTR2 without (open squares) and with (open triangles) cross-linking. Data for KMTR2 and the LkN53R mutant are shown as solid and dashed lines, respectively. (**b**) The effector caspase activity of Colo205 was assessed 2 h after treatment with a monomer fraction of KMTR2, LkN53R, and aDNPG1 (negative control) without (dark gray) and with (light gray) cross-linking.

We examined whether the LkN53R mutant resulted in a loss of apoptosis-inducing activity without cross-linking. KMTR2 exhibited concentration-dependent inhibition of the proliferation of Colo205 cells (EC_50_ = 6–7 ng/mL) (Fig. 5a) even in the absence of a cross-linking reagent. In contrast, the LkN53R mutant exhibited no inhibitory activity at any concentration. However, both antibodies induced apoptosis in a concentration-dependent manner and with similar activities in the presence of a cross-linking regent, although the activity of the LkN53R mutant was less than that of KMTR2 even if a cross-linking reagent was used (Fig. 5a; KMTR2: EC_50_ = 4–5 ng/mL; LkN53R: EC_50_ = 10–20 ng/mL). In addition, the IgG monomer of KMTR2 induced marked caspase-3/7 activation of the same level as cross-linked KMTR2 at concentrations of 100 and 1,000 ng/mL (Fig. 5b). In contrast, the LkN53R mutant exhibited caspase-3/7 activation only when the cross-linking reagent was added. These results indicated that the LkN53R mutant did not have agonistic activity without cross-linking and strongly suggested that the LkN53R mutant interfered with KMTR2 Fab dimerization, which resulted in a loss of its direct agonistic activity.

## Discussion

Our results suggested that the 2:2 configuration shown in Fig. 2a was an essential unit for sufficient KMTR2 direct agonistic activity. In the KMTR2-Fab structure without TRAIL-R2, CDR2 of the KMTR2 light chain was also involved in the interaction with neighboring molecules via crystallographic symmetry (Fig. S3). There were some interaction residues between each CDR2 of the light chain; however, its binding mode was quite different from that in complex with TRAIL-R2. In the presence of TRAIL-R2, the unique sequence at CDR2 of the light chain in KMTR2 was responsible for its association with KMTR2, which resulted in effective superoligomerization and activation of potent apoptotic signaling via TRAIL-R2.

**Figure 6:**
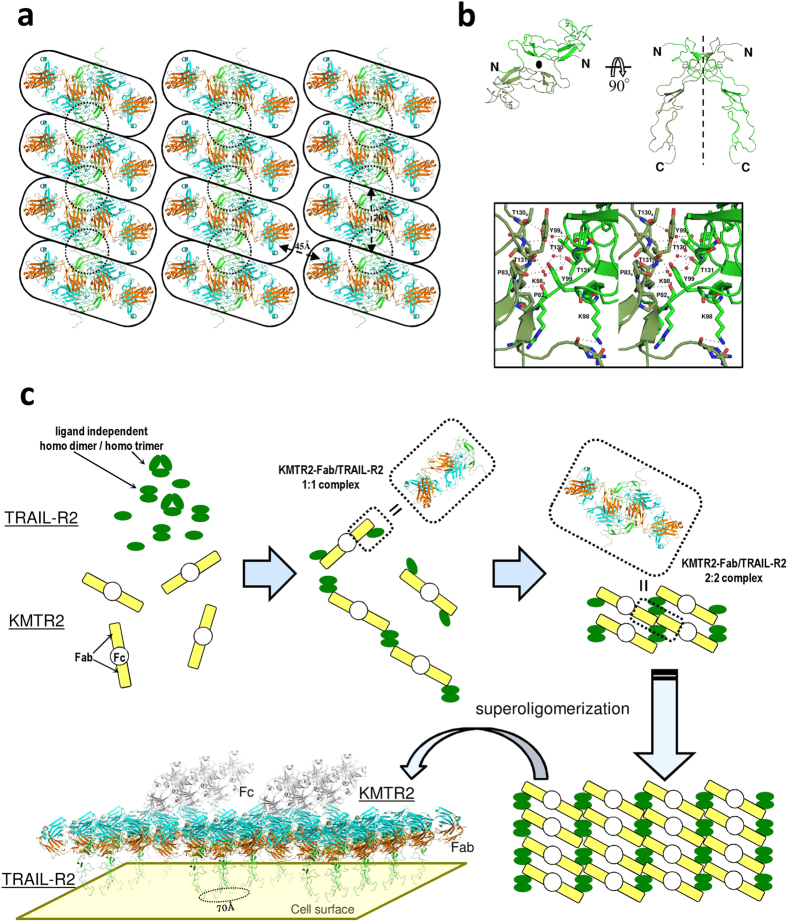
TRAIL-R2 oligomerization induced by KMTR2. (**a**) Oligomeric structure of an ecTRAIL-R2/KMTR2-Fab complex on a layer in a crystal lattice. The 2:2 complexes and TRAIL-R2 dimers rendered by another two-fold symmetry are enclosed by rounded rectangles and dotted circles, respectively. (**b**) The TRAIL-R2 dimer enclosed by a dotted circle in (**a**). A close-up view of the interface between two TRAIL-R2 molecules is drawn in the boxed figure (stereo representation). Residues related to the interaction between two TRAIL-R2 molecules are represented by a stick model. (**c**) Model for TRAIL-R2 superoligomerization by KMTR2. The intact model structure of KMTR2 that was constructed with reference to the crystal structure of an intact IgG2a monoclonal antibody (PDB ID: 1IGT)^39^.

Our previous results using SEC-light scattering analysis showed that the molecular mass of the ecTRAIL-R2:Fc/KMTR2 complex eluted in the void volume fraction (Fig. 4a, upper) was 5,130 kDa^24^. This mass corresponds to approximately twenty 2:2 complexes between KMTR2 and ecTRAIL-R2 (Fc regions of the antibody and antigen were included in this calculation). We assumed that TRAIL-R2 superoligomerization induced by KMTR2 would appear in its crystallographic packing. Several 2:2 complexes on the same layer in a crystal lattice were extracted (Fig. 6a). In the vertical direction, each 2:2 complex (shown within rounded rectangles) was linked with another unit of the 2:2 complex via an interface between two TRAIL-R2 molecules (shown within dotted circles).

It is known that many receptors belonging to the TNF receptor superfamily exist as preassembled oligomers on a cell surface^27^,^28^. TRAIL-R2 also generates a ligand-independent assembly with TRAIL-R4, which acts as a decoy receptor^29^. TNF receptor-1 and -2 form a self-complex of homodimers or homotrimers as preassembled oligomers on a cell surface^27^,^30^. During the ligand-independent assembly of TNF receptors, each receptor associates with others via CRD1^27^, and a parallel dimer of TNF receptor-1 through a CRD1–CRD1 interaction has been confirmed in its crystal structure (Fig. S4)^31^. A similar CRD1–CRD1 interaction was also observed in the crystal packing between TRAIL-R2 molecules (Fig. 6b), in which the CRD1–CRD1 interface was constructed via two-fold crystallographic symmetry (shown within dotted circles in Fig. 6a). The side chain of Lys98 interacted with two main chain carbonyl oxygen atoms in neighboring symmetric molecule by hydrogen bonding. The aromatic ring of Tyr99 and two sequential prolines (Pro82 and Pro83) in the neighboring molecule were situated in positions permitting van der Waals interaction. In addition, two sequential threonine residues (Thr130 and Thr131) formed a hydrogen bond network with identical residues in the neighboring molecule via several water molecules. The mutation study of TNF receptor-1 showed that the serial Lys–Tyr residues (Lys19 and Tyr20) in CRD-1 were essential for the ligand-independent self-association and signal transduction^27^ (however, in the crystal structure^31^, Tyr20 was located far from the neighboring molecule, as shown in Fig. S4). This information suggests that the symmetric TRAIL-R2 dimer may be a ligand-independent homodimer.

These findings suggest that KMTR2 acts as a bridge between preassembled TRAIL-R2 dimers and results in the linear oligomerization observed in its crystal packing, in which the distance between each TRAIL-R2 dimer is approximately 70 Å. This distance corresponds to that in both the “dimer” and “trimer” models of TNF receptor-2 proposed as the signaling oligomers of TNF/TNF receptor-2 complexes^30^. With respect to the adjacent relationship between the linear packing of ecTRAIL-R2/KMTR2, there is no direct packing interaction between 2:2 complexes (Fig. 6a). The distances between the C-terminals of the KMTR2-Fab heavy chain in this linear packing are, however, approximately 45 Å apart (Fig. 6a). Because the distance between the C terminals of Fab was 45 Å in the crystal structure of IgG, this distance (45 Å) enables these two Fab fragments to exist as two “arms” in one intact IgG molecule.

Based on these considerations, we propose the clustering mechanism of TRAIL-R2 provided by KMTR2 (Fig. 6c). Although the enhanced clustering (trimer model) of TRAIL-R2 is proposed from the structure of the TRAIL/TRAIL-R2/AMG 655-Fab ternary complex^15^, KMTR2 constructs such clustering without TRAIL and consequently shows a strong direct agonistic activity. The schematic clustering model drawn in Fig. 6c shows that four TRAIL-R2 molecules localize in parallel within 70 Å on cell surface. Such conformation of receptors may allow the adjacent positioning of the intracellular death domains (DDs) sufficient for activating the caspase-dependent apoptotic pathway. In crystal structures of the death-inducing signaling complex (DISC) formed by Fas receptor and Fas-associaetd death domain protein, 4^32^ and 5–7^33^ Fas-DDs composed the DISC, which activates caspases. It is believed that superoligomerization of receptors with methodological clustering (~70 Å and parallel direction) induced by KMTR2 is essential for transducing an apoptosis signal.

## Methods

### ecTRAIL-R2 and mutant preparation

The extracellular region (residues Ala54–Lys181) of human TRAIL-R2 fused with the Fc region of human IgG1 was expressed in CHO cells. A FLAG sequence (DYKDDDDK) was inserted between ecTRAIL-R2 and Fc. The ecTRAIL-R2:Fc fusion protein was purified using a HiTrap rProtein A FF column (GE Healthcare, Piscataway, NJ) for affinity chromatography and then digested using a recombinant enterokinase cleavage kit (Novagen) according to the supplier’s protocol. ecTRAIL-R2 and Fc were purified by cation-exchange chromatography on a TSKgel SP-5PW column (4.5 × 75 mm, TOSOH, Tokyo, Japan) after removing enterokinase with capture agarose. ecTRAIL-R2 and Fc were separated using a linear gradient of 0–0.5 M NaCl in 20 mM sodium acetate (pH 5.0).

### KMTR2 and mutant preparation

KMTR2 expression and purification were performed as reported previously^24^. Single amino acid substitution (Asn53 → Arg) in the light kappa chain of KMTR2 was introduced using the primers 5′-GAT GCA TCC AGA AGG GCC ACT GG-3′ and 5′-CCA GTG GCC CTT CTG GAT GCA TC-3′. Site-directed mutagenesis was conducted using a N5KG4-KMTR2 vector^24^ and a QuikChange site-directed mutagenesis kit (Stratagene, La Jolla, CA) according to the supplier’s protocol. After site-directed mutagenesis, the N5KG4-KMTR2 vector with substituted Asn53 to Arg in the light kappa chain (LkN53R) was transformed in *Escherichia coli* DH5α cells, and the LkN53R mutant was expressed and purified using the same procedures as used for KMTR2^24^.

An anti-TRAIL-R2 fully human monoclonal antibody KMTR2 was recombinantly prepared as reported previously^24^. A Fab fragment was prepared by papain (Boehringer Ingelheim, Germany) digestion at the hinge region of KMTR2 to remove the Fc region. Antibodies were incubated for 1 h under both non-reducing and reducing (10 mM cysteine) conditions, after which they were incubated with activated papain for 24 h. Fab and Fc fragments were separated by cation-exchange chromatography on a TSKgel SP-5PW column using a linear gradient of 0–0.5 M NaCl in 20 mM sodium acetate (pH 5.0).

### ecTRAIL-R2/KMTR2-Fab complex preparation

ecTRAIL-R2 was mixed with an excess amount of KMTR2-Fab and incubated at room temperature (RT) for 1 h to form the ecTRAIL-R2/KMTR2-Fab complex. The ecTRAIL-R2/KMTR2-Fab complex was purified by gel filtration using a Superdex 200 HR 10/30 column (Amersham Pharmacia Biotech AB, Uppsala, Sweden) equilibrated with 20 mM HEPES buffer (pH 7.2) with 0.2 M NaCl at a flow rate of 0.3 mL/min. The observed mass of the eluted complex by multi-angle light scattering using mini-DAWN (Wyatt Technologies, Santa Barbara, CA) indicated that ecTRAIL-R2 and KMTR2-Fab had formed a 1:1 stoichiometric complex.

### Crystallization

Purified ecTRAIL-R2/KMTR2-Fab complexes and KMTR2-Fab fragments were concentrated to 5 mg/mL in 20 mM HEPES buffer (pH 7.2) that contained 0.2 M NaCl. Initial screening of crystallization conditions was performed using Crystal Screen 1 & 2 and PEG/Ion Screen 1 & 2 (Hampton Research, Riverside, CA) by vapor diffusion against the reservoir solution. Microcrystals of ecTRAIL-R2/KMTR2-Fab were obtained from condition 7 in PEG/Ion Screen 1. Crystallization conditions were finally refined to 75 mM calcium chloride dihydrate (pH 5.1) containing 7.5% (w/v) PEG3350 to yield prism-shaped crystals with a dimension of approximately 0.02 × 0.04 × 0.15 mm. Prism-shaped crystals of KMTR2-Fab with a dimension of approximately 0.05 × 0.05 × 0.2 mm were obtained from condition 33 [200 mM sodium sulfate decahydrate, pH 6.6, with 20% (w/v) PEG3350] in PEG/Ion Screen 1 after the initial crystallization screening.

### Data collection, phasing, and refinement

Diffraction data for ecTRAIL-R2/KMTR2-Fab complexes and KMTR2-Fab fragments were acquired in a cold nitrogen gas stream at 100 K and recorded on an ADSC Quantum 315 at BL41XU at the SPring-8 facility (Hyogo, Japan), with a total oscillation range of 180°. The oscillation angle and exposure time per frame were 1.0° and 10 s, respectively. Intensity data for ecTRAIL-R2/KMTR2-Fab complexes and KMTR2-Fab fragments were processed with HKL2000^34^ to 2.1 Å and 2.5 Å resolution, respectively. The crystals of ecTRAIL-R2/KMTR2-Fab complexes and KMTR2-Fab fragments belonged to space group *I*222 with unit cell dimensions of *a* = 145, *b* = 152, and *c* = 65 Å and *C*222_1_ with unit cell dimensions of *a* = 153, *b* = 165, and *c* = 66 Å, respectively. The Matthews coefficients of ecTRAIL-R2/KMTR2-Fab complexes and KMTR2-Fab fragments were 2.77 Å^3^/Da and 4.26 Å^3^/Da assuming one 1:1 complex and Fab in the asymmetric unit corresponding to solvent contents of 56% and 71%, respectively. Initial phase information was obtained by molecular replacement analysis with PHASER^35^ using a Fab structure of human IgG1 anti-HIV-1 (PDB ID: 1HZH)^36^ and a structure of an ecTRAIL-R2 in 1:1 complex structure with TRAIL (PDB ID: 1D4V)^11^ as search models. The atomic model was built and modified with COOT^37^. The final models for ecTRAIL-R2/KMTR2-Fab complexes and KMTR2-Fab fragments were refined to crystallographic *R*-factors of 18.8% and 18.8% (free *R*-factors = 22.4% and 22.4%) to 2.1 Å and 2.5 Å resolution using TLS and restrained refinement with REFMAC^38^, respectively. Statistics for data collection and refinement are summarized in Table S1. Figs 1, 2 and 6, and S1–S4 were prepared using the PyMOL Molecular Graphics System (DeLano Scientific, San Carlos, CA, USA).

### Flow cytometry

The human colorectal adenocarcinoma cell line Colo205 (ATCC No. CCL-222) was cultured in RPMI 1640 medium supplemented with 10% fatal bovine serum (FBS) in 96-well round-bottomed plates (BD Biosciences PharMingen, San Jose, CA) at a density of 5 × 10^5^ cells/well. KMTR2, LkN53R mutant, and an anti-dinitrophenol (DNP) monoclonal antibody IgG1 (aDNPG1) as a negative control were added to wells at concentrations of 100 or 1000 ng/mL and then incubated with R-phycoerythrin (RPE)-labeled goat anti-human IgG (Southern biotech) at 4 °C for 1 h. After washing, the cells were analyzed using a FACSCalibur (BD Biosciences PharMingen), as described previously^23^.

### ELISA

Purified ecTRAIL-R2:Fc fusion proteins were added to a flat-bottomed plate at 25 ng/well and incubated at RT for 1 h. The wells were then blocked with blocking buffer (Pierce, Rockford, IL) at RT for 30 min. After three washes with wash buffer (Tris-buffered saline with 0.1% Tween-20), KMTR2, KMTR2 mutants, and aDNPG1 as negative control were added to the wells at concentrations of 2–1000 ng/mL and incubated at RT for 1 h. The plate was washed three times with wash buffer; thereafter, a horseradish peroxidase-labeled secondary antibody, goat anti-human kappa polyclonal antibody (dilution 1:2000; Biosource International), was added to each well, followed by incubation at RT for 1 h. The plate was washed three times with wash buffer, after which TMB substrate (DAKO Japan, Kyoto, Japan) was added. The enzymatic reaction was stopped by adding 0.5 mol/L of sulfonic acid and absorbance was measured at 450 nm (reference wavelength of 570 nm) using a microplate reader.

### Size exclusion chromatography

KMTR2 and the LkN53R mutant antibodies were mixed with ecTRAIL-R2:Fc in PBS at a molar ratio of 1:1. The mixture was incubated for 30 min at 37°C and then loaded onto Superdex 200 10/300 GL gel filtration column (GE Healthcare, Piscataway, NJ) equilibrated with D-PBS(-). The protein complexes of each fraction were detected by UV absorbance at 280 nm.

### Confocal microscopic analysis

Colo205 cells were seeded in collagen I two-well culture slides (BD Biosciences PharMingen, San Jose, CA) at 2 × 10^5^ cells per well and cultured overnight at 37 °C under 5% CO_2_. The cells were incubated with gel filtration fractionated monomeric KMTR2 or the LkN53R mutant at a concentration of 100 ng/mL for 1.5 h at 37 °C under 5% CO_2_. After washing with ice-cold D-PBS(-) containing 1% FBS, Alexa 488-labeled Fab fragment specifically recognizing human IgG (Zenon human IgG labeling kits, Molecular Probes) was added for 30 min at 4 °C. The cells were washed and mounted in Fluoromount-G (Southern-Biotech, Birmingham, AL) and visualized using a confocal microscope (Carl Zeiss, Oberkochen, Germany) with a ×40 objective lens.

### Proliferation assay

Colo205 cells were seeded in a 96-well flat-bottom plate at a density of 1 × 10^4^ cells/well and cultured overnight at 37 °C under 5% CO_2_. KMTR2 and LkN53R mutant antibodies were added to the wells at concentrations of 1–100 ng/mL, after which the cells were cultured for an additional 2 days. Cross-linked KMTR2 was prepared by adding goat anti-human IgG (gamma-chain specific; Sigma-Aldrich) at a concentration of 10 μg/mLfor 0.5–1 h after adding KMTR2. Cell viability was determined by an MTS dye reduction assay using a CellTiter 96 aqueous non-radioactive cell proliferation assay kit (Promega Corp., Madison, WI) and was calculated as follows:


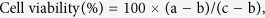


where a = absorbance of a sample well, b = absorbance of a blank well, and c = absorbance of medium-treated cells. Each measurement was repeated three times with similar results. The EC_50_ value for each antibody was defined as the concentration that reduced cell viability by 50%.

### Caspase assay

Colo205 cells were seeded in a 96-well flat-bottom plate (Corning Life Sciences, Corning, NY) at 1 × 10^4^ cells per well and cultured overnight at 37 °C under 5% CO_2_. KMTR2, LkN53R mutant, and aDNPG1 (as negative control) antibodies were added to each well at 1, 10, 100, and 1,000 ng/mL (aDNPG1: 1,000 ng/mL only)and incubated for 8 h. Cells were added with or without goat anti-human IgG at a concentration of 10 μg/mL. Two hours after the addition of antibodies, caspase-3/7 activity was measured using the Apo-ONE homogeneous caspase-3/7 assay kit (Promega Corp., Madison, WI).

## Additional Information

**Accession codes:** The atomic coordinate and structure factors for TRAIL-R2/KMTR2-Fab complexes and KMTR2-Fab fragments have been deposited in the Protein Data Bank with accession numbers 3X3F and 3X3G, respectively.

**How to cite this article**: Tamada, T. *et al.* TRAIL-R2 Superoligomerization Induced by Human Monoclonal Agonistic Antibody KMTR2. *Sci. Rep.* **5**, 17936; doi: 10.1038/srep17936 (2015).

## Supplementary Material

Supplementary Information

## Figures and Tables

**Table 1:** Residues related to antigen–antibody binding.

TRAIL-R2					KMTR2			
epitope	residue	atom	distance		atom	residue	CDR	chain
1	Ilu87	Cγ2	3.73		Cβ	Trp94	3	L
	Glu89	O	2.96	h	Nε1	Trp50	2	H
		Cδ	3.89		Cε	Lys33	1	
		Oε1	3.07	h	Nζ			
			2.72	h	N	Gly103	3	
		Oε2	3.24	h	Nζ	Lys33	1	
			water mediated	h	Nε1	Trp50	2	
			water mediated	h	N	Asn52		
			water mediated	h	Oγ	Ser57		
	Asp90	N	water mediated	h	O	Thr58		
		Cβ	3.84		Cα	Gly59		
		C	3.91		Cε2	Trp94	3	L
	Gly91	Cα	3.61		Cδ1			
2	Asp102	Oδ2	2.97	h	Nη1	Arg107	3	H
			water mediated	h	Nη2			
	His106	Nε2	water mediated	h	Nε2	Gln27	1	L
			water mediated	h	Nδ2	Asm93	3	
	Asp109	N	water mediated	h				
		O	2.85	h	N	Trp94		
		Oδ1	2.65	h	Nδ2	Asn93		
		Oδ2	water mediated	h	O	Trp94		
			water mediated	h	N	Glu1	─	
	Leu111	N	2.87	h	O	Ser92	3	
		Cβ	3.96		Cε1	Tyr106	3	H
		Cδ1	3.82		Cβ	Trp94	3	L
		Cδ2	3.67		C	Asn93		
			3.70		Cδ1	Leu96		
	Phe112	Cδ1	3.71		Cδ1	Tyr106	3	H
			3.55		Cβ	Arg107		
		Cε2	3.52		Cε1	Tyr32	1	L
		Cζ	3.73		Cα	Ser92	3	
	Cys113	O	2.78	h	Nη1	Arg107	3	H
	Arg115	N	water mediated	h	Oη	Tyr32	1	L
		Cγ	3.27		Cζ	Arg107	3	H
		Nη1	3.24	h	Oδ2	Arg108		
			water mediated	h	Oη	Tyr32	1	L
			water mediated	h	O	Arg107	3	H

h: hydrogen bond (within 3.6 Å between donor and acceptor).

**Table 2:** Residues related to antibody–antibody binding via crystallographic symmetry.

						Symmetry			
chain	CDR	residue	atom	distance		atom	residue	CDR	chain
H	1	Gly26	O	3.50	h	Oε1	Glu1	─	H
L	2	Tyr49	Oη	2.59	h	O	Arg54	2	L
		Asp50	Oδ2	water mediated	h	Nη1			
			O	water mediated	h	Nη2			
		Ser52	Oγ	water mediated	h	O	Ser52		
		Asn53	Oδ1	2.90	h	N	Arg54		
			Nδ2	water mediated	h	Nη2			
		Arg54	Nη1	3.59	h	O	Asp108	3	H
				water mediated	h	O	Tyr110		
				water mediated	h	Nη1	Arg91	3	L
		Thr56	Cα	3.95		Cδ2	Tyr111	3	H
		Gly57	Cα	3.47		Cζ			
			N	3.12	h	Oη			
		Ile58	O	2.75	h	Oη			
		Pro59	Cβ	3.52		Cζ	Tyr109		
		Ala60	N	2.99	h	O	Asp108		
			Cβ	3.99		Cα			
		Arg61	Nη1	water mediated	h	Oδ1			
				water mediated	h	Oη1	Tyr109		

h: hydrogen bond (within 3.6 Å between donor and acceptor).
