# Supplementary material for: TRAIL-R2 Superoligomerization Induced by Human Monoclonal Agonistic Antibody KMTR2
Source: Sci Rep. 2015 Dec 17;5:17936. doi: 10.1038/srep17936 (PMC4682084; doi:10.1038/srep17936)
Supplement: Supplementary Information [file srep17936-s1.pdf]

Supplementary information

TRAIL-R2 Superoligomerization Induced by Human Monoclonal Agonistic Antibody KMTR2

Taro Tamada[1], Daisuke Shinmi[2], Masahiro Ikeda[3], Yasushi Yonezawa[1], Shiro Kataoka[4], Ryota Kuroki[1,#], Eiji Mori[5], Kazuhiro Motoki[6]

[1]Quantum Beam Science Center, Japan Atomic Energy Agency, 2-4, Shirakata, Tokai, Ibaraki 319-1195, Japan.

[2]Research Core Function Laboratories, R&D Division, Kyowa Hakko Kirin Co., Ltd., 3-6-6, Asahi-machi, Machida, Tokyo, 194-8533, Japan.

[3]Immunology & Allergy Research Laboratories, R&D Division, Kyowa Hakko Kirin Co., Ltd., 3-6-6, Asahi-machi, Machida, Tokyo, 194-8533, Japan.

[4]Bussiness Development Department, Kyowa Hakko Kirin Co., Ltd., 1-6-1, Ohtemachi, Chiyoda-ku, Tokyo, 100-8185, Japan.

[5]R&D Planning Department, R&D Division, Kyowa Hakko Kirin Co., Ltd., 1-6-1, Ohtemachi, Chiyoda-ku, Tokyo, 100-8185, Japan.

[6]Oncology Research Laboratories, R&D Division, Kyowa Hakko Kirin Co., Ltd. 3-6-6, Asahi-machi, Machida, Tokyo, 194-8533, Japan.

#Deceased

Correspondence and requests for materials should be addressed to: T.T. (e-mail: tamada.taro@jaea.go.jp).

Table S1. Data collection and refinement statistics.

	**ecTRAIL-R2/KMTR2-Fab**	**KMTR2-Fab**
**Crystal Data**		
Space group	*I*222	$C222_1$
Cell constants (Å)	*a* = 145, *b* = 152, *c* = 65.0	*a* = 153, *b* = 165, *c* = 65.3
**Data Collection**		
Resolution (Å)	46.73–2.10 (2.18–2.10)	38.25–2.50 (2.59–2.50)
Reflections (total/unique)	195,258/39,887	173,069/27,370
Redundancy	4.9 (2.7)	6.3 (3.9)
Mosaicity	0.54	0.38
$I/\sigma(I)$	12.1 (1.9)	27.6 (3.8)
R_{merge}[1]	0.080 (0.294)	0.063 (0.396)
Completeness (%)	94.9 (84.2)	95.4 (81.1)
**Refinement**		
Resolution (Å)	46.73–2.10 (2.16–2.10)	38.25–2.51 (2.58–2.51)
Used reflections	39,887	27,361
R_{cryst}[2]	0.188 (0.285)	0.188 (0.321)
R_{free}[3]	0.224 (0.323)	0.224 (0.377)
No. of atoms	4,199	3,418
protein	3,878	3,349
sugar chain	14	14
glycerol, ion	31	13
water	276	42
Mean B value (Å^2)	32.8	78.27
ecTRAIL-R2	37.2	–
KMTR2-Fab heavy chain	32.7	74.2
KMTR2-Fab light chain	30.6	82.4
r.m.s. deviation		
bond lengths (Å)	0.008	0.012
bond angle (°)	1.36	1.70

Values for highest resolution shells are shown in parentheses.

[1]$R_{merge} = \Sigma|I(\boldsymbol{h}) - \langle I(\boldsymbol{h})\rangle|/\Sigma I(\boldsymbol{h})$, where $\langle I(\boldsymbol{h})\rangle$ is the mean value of reflection $\boldsymbol{h}$ for all measurements of $I(\boldsymbol{h})$.

[2]$R_{cryst} = \Sigma||F_{obs}| - |F_{calc}||/\Sigma|F_{obs}|$, where F_{obs} and F_{calc} are observed and calculated structure factor amplitudes, respectively.

[3]R_{free} is the same as R_{cryst}, except for a 5% subset of all reflections.

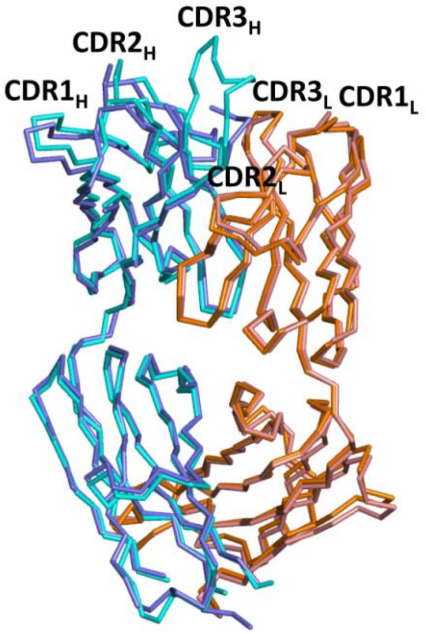

Fig. S1. Structural comparison of KMTR2-Fab with (heavy: cyan, light: orange) and without (heavy: purple, light: beige) TRAIL-R2. The two structures were superimposed using all main chain atoms.

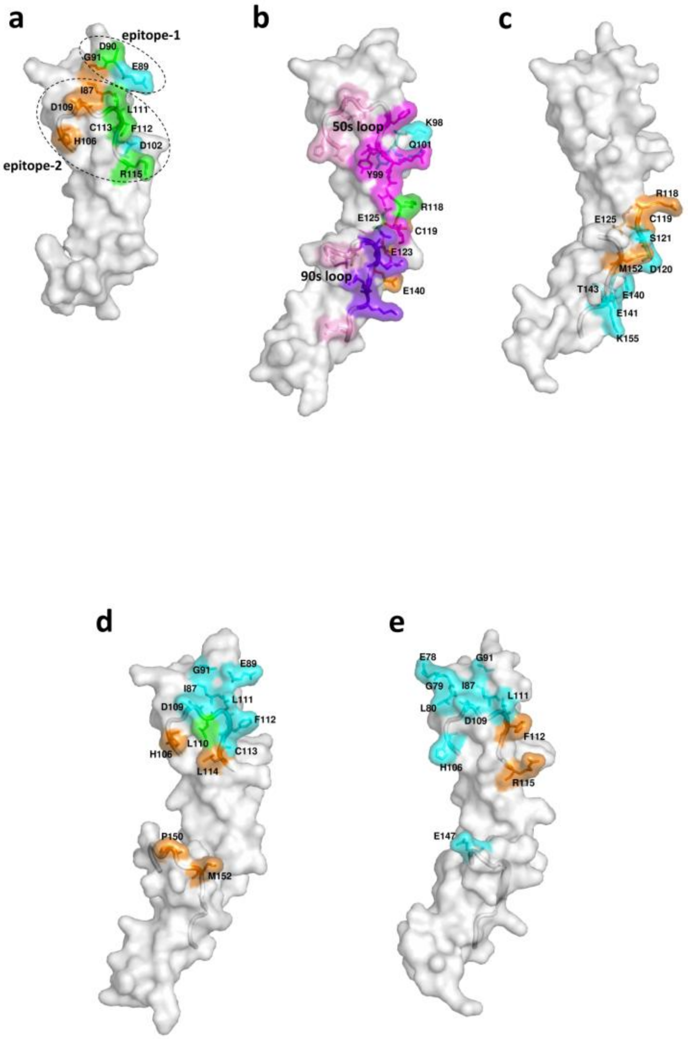

Fig. S2. Comparison of the structural epitopes of TRAIL-R2 for binding to (a) KMTR2, (b) AMG 655, (c) Apomab, (d) BDF1, and (e) YSd1. ecTRAIL-R2 is shown as a molecular surface model. Residues recognized by light, heavy, and both chains of Fab are drawn as a stick model and colored orange, cyan, and green, respectively. Residues recognized by TRAIL are colored in magenta (by TRAIL molecule in 1:1 complex), pink (by TRAIL molecule in neighbor complex), and purple (by both TRAIL molecules) in (b).

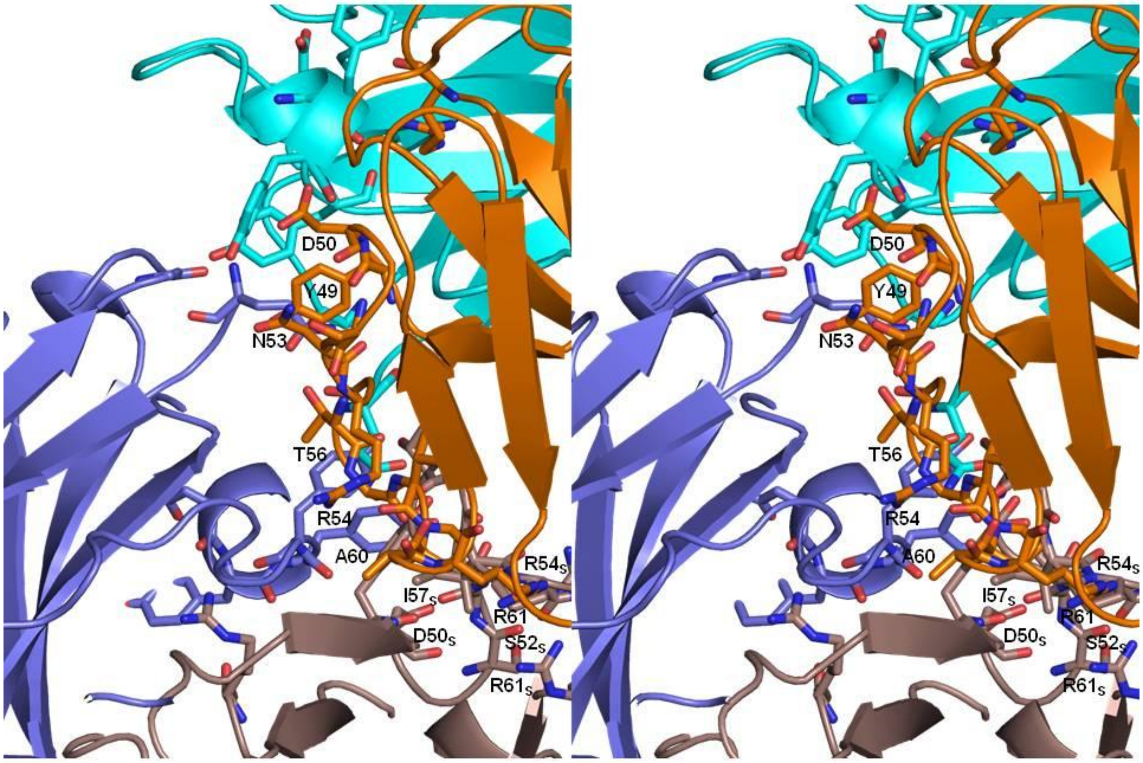

Fig. S3. Close-up view of the interface between two KMTR2-Fab molecules in a structure without ecTRAIL-R2 rendered by crystallographic symmetry (stereo representation). KMTR2-Fab molecule (heavy: cyan, light: orange) at right is shown in the same position as that in Fig. 2b. The subscript "S" in label means "symmetry" molecule.

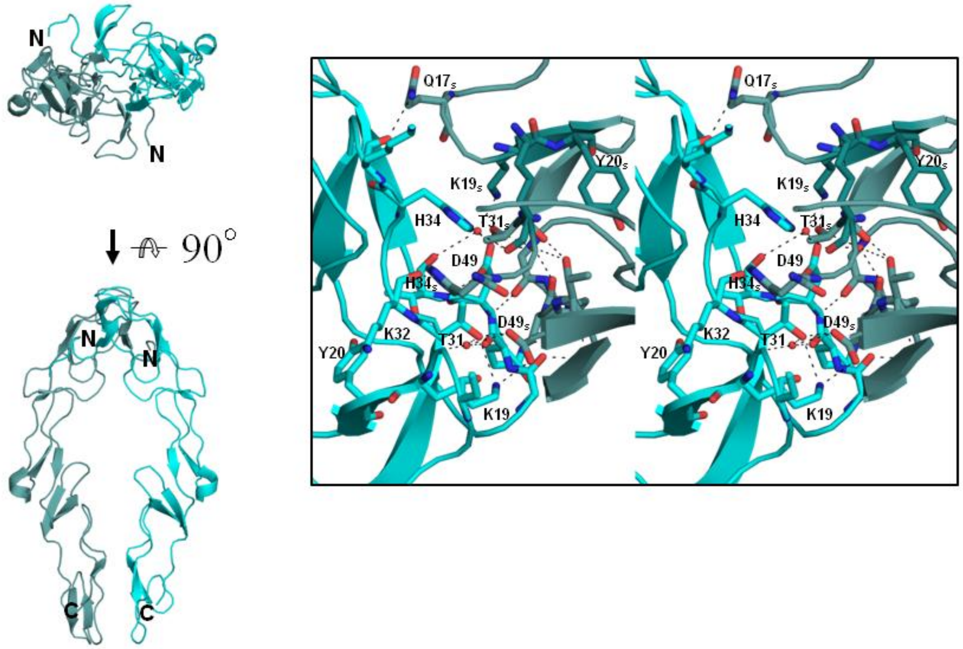

Fig. S4. Crystal structure of TNR receptor-1 dimer. A close-up view of the interface between two receptor molecules is drawn in the boxed figure (stereo representation).
